# Advances in the Study of Bionic Mineralized Collagen, PLGA, Magnesium Ionomer Materials, and Their Composite Scaffolds for Bone Defect Treatment

**DOI:** 10.3390/jfb14080406

**Published:** 2023-08-01

**Authors:** Shuai Zhou, Shihang Liu, Yan Wang, Wenjing Li, Juan Wang, Xiumei Wang, Shuo Wang, Wei Chen, Hongzhi Lv

**Affiliations:** 1 Department of Orthopaedic Surgery, The Third Hospital of Hebei Medical University, No. 139 Ziqiang Road, Shijiazhuang 050051, China; masterjoe24@163.com (S.Z.); 13831974652@163.com (S.L.); 18810310994@163.com (Y.W.); m17865583127@163.com (W.L.);; 2 Key Laboratory of Biomechanics of Hebei Province, Orthopaedic Research Institution of Hebei Province, No. 139 Ziqiang Road, Shijiazhuang 050051, China; 3 NHC Key Laboratory of Intelligent Orthopaedic Equipment, The Third Hospital of Hebei Medical University, No. 139 Ziqiang Road, Shijiazhuang 050051, China; 4 State Key Laboratory of New Ceramics and Fine Processing, School of Materials Science and Engineering, Tsinghua University, No. 30 Shuangqing Road, Beijing 100084, China

**Keywords:** bone defect treatment, mineralized collagen, PLGA, magnesium, composite scaffold, bone regeneration

## Abstract

The healing of bone defects after a fracture remains a key issue to be addressed. Globally, more than 20 million patients experience bone defects annually. Among all artificial bone repair materials that can aid healing, implantable scaffolds made from a mineralized collagen (MC) base have the strongest bionic properties. The MC/PLGA scaffold, created by adding Poly (lactic-co-glycolic acid) copolymer (PLGA) and magnesium metal to the MC substrate, plays a powerful role in promoting fracture healing because, on the one hand, it has good biocompatibility similar to that of MC; on the other hand, the addition of PLGA provides the scaffold with an interconnected porous structure, and the addition of magnesium allows the scaffold to perform anti-inflammatory, osteogenic, and angiogenic activities. Using the latest 3D printing technology for scaffold fabrication, it is possible to model the scaffold in advance according to the requirement and produce a therapeutic scaffold suitable for various bone-defect shapes with less time and effort, which can promote bone tissue healing and regeneration to the maximum extent. This study reviews the material selection and technical preparation of MC/PLGA scaffolds, and the progress of their research on bone defect treatment.

## 1. Introduction

The incidence of fractures in the Chinese population is gradually increasing and now exceeds 5 million people/year [1,2,3], with approximately 5–10% of fracture patients experiencing delayed healing or bone discontinuity annually [4,5], resulting in a serious decline in patients’ quality of life. Globally, more than 20 million patients experience bone defects annually, and the average treatment cost per person is much higher than USD 5000 [6,7]. Therefore, fracture healing disorders and bone defect repair have become important and difficult areas for orthopedic treatments. The clinical efficacy of fracture healing treatment techniques such as autologous bone grafting are relatively clear; however, they are highly invasive, costly, and have a high likelihood of non-healing after surgery [8,9]. Non-operative treatments such as hyperbaric oxygen and blood transfusions have shown some therapeutic effects; however, they are less targeted, have a longer treatment period, and can increase patient pain [10,11,12]. Therefore, further insight into the fracture healing mechanism and the development of technologies and products that can promote osteogenesis in a controlled manner while considering the physiological state of the patient and reducing the effects of injury or adverse effects are key to shortening the bone repair cycle, promoting the avoidance of delayed fracture healing or clinical efficacy of bone nonunion, and improving patient outcomes. Therefore, the development of an ideal artificial bone graft material is gradually becoming a research priority.

Mineralized collagen (MC)-based materials have the best bionic properties among all the tissue-engineered bone materials. Bone tissue is a natural nanocomposite composed of 60% minerals, 30% matrix, and 10% water [13]. The main component of the bone matrix is collagen, which provides toughness to the bone tissue. The mineral composition of the bone is mainly non-chemo ratio hydroxyapatite (HA), which combines sodium, magnesium, and potassium ions and provides stiffness to the bone tissue [14]. Therefore, to mimic the composition and nanostructure of natural bone, a bone-defect scaffold based on MC was created. Studies have shown that MC scaffolds have good biocompatibility, osteoconductivity, and osteogenic properties, can be compounded with other artificial bone materials and metal ions, and can be used as carriers for delivering drugs and growth factors, which have a wide range of applications in bone repair materials [15,16]. However, poor mechanical properties limit the further application of MC in load-bearing bone defects. Therefore, modifications are required to obtain tissue-engineered scaffolds with higher mechanical strengths.

Polylactic acid–hydroxyacetic acid copolymers (PLGAs) are biocompatible and degradable [17]. Moreover, their mechanical strength and degradation rate can be controlled artificially [8]. These properties make PLGAs excellent for medical applications [18,19,20]. Studies have shown that PLGAs improve the mechanical and osteogenic properties of MC materials [21]. Magnesium (Mg) is one of the main elements in bone tissue and is involved in a variety of cellular processes and functions, inhibiting the inflammatory response of macrophages and accelerating osteoblast differentiation and mineralization. Therefore, Mg^2+^ was added to the MC/PLGA scaffold to investigate the binding role of Mg to MC/PLGAs during fracture healing. The function of the regenerated bone tissue is highly dependent on the three-dimensional (3D) scaffold structure. An ideal 3D scaffold should have an interconnected pore structure that provides space for cell adhesion and vascular ingrowth and transports nutrients and metabolic waste while maintaining its mechanical properties. 3D printing technology is an effective method to maintain the structural integrity and stability of the scaffold, allowing the material to be fabricated into any desired shape of prosthetic bone based on a variety of complex bone defects, and the use of 3D printing technology to prepare MC/PLGA scaffolds is a reliable method.

At present, there is limited research on MC/PLGA scaffolds doped with Mg ions, which are relatively novel and theoretically an ideal material for treating bone defects. The purpose of writing this review is to provide more innovative research and ideas on new materials for repairing bone defects.

In summary, this paper will review the progress of MC/PLGA material preparation and treatment for repairing bone defects.

## 2. Ideal Materials and Their Preparation for Repairing Bone Defects

### 2.1. Bone Defects and Commonly Used Bone Repair Materials

Bone defects are congenital or acquired causes of bone matrix shortages and a disruption of bone structure integrity, resulting in non-healing or delayed bone healing and localized physical dysfunction [22]. Annually, more than two million patients worldwide undergo bone graft surgery for bone defects. With the increasing incidence of fractures in Chinese patients, the number of patients with bone defects is also increasing annually [1,2,23,24,25]. Bone defects are difficult to treat, require large amounts of bone tissue to fill, have many postoperative complications, and are prone to medical disputes, making them one of the most common and difficult-to-treat defects in clinical settings [26,27,28].

Autologous bone flap grafting with vascular tips is the gold standard for bone defect treatment; however, it is prone to complications such as secondary injury to the donor area, and its source is limited [8]. Allogeneic bone tissue filling is a common treatment for bone defects; however, it lacks blood supply and carries rejection and infection risks [29]. Owing to the urgent clinical need to develop a bone repair material with structural and functional similarities to natural bone and good biocompatibility, bone tissue engineering has emerged and developed rapidly in recent decades [30]. The developed bone repair material is implanted into the defect site to repair or reconstruct the bone defect by providing a suitable environment for cells and growth factors to exert their physiological effects and promote new bone tissue growth [31]. The three main categories of commonly used bone repair materials are natural, synthetic, and metallic [32].

Chitosan, collagen, fibrin, and other materials produced by living organisms are natural biomaterials with a wide range of sources, good biocompatibility, and low immunogenicity [33]. Collagen, an organic component of natural bone tissue, is a natural polymer widely used in biomedical applications. However, pure collagen has poor compression resistance, and many researchers have combined collagen with materials such as apatite crystals to create composite scaffolds with improved mechanical properties [34,35]. The synthetic materials include organic and inorganic bioceramics. Organic materials, such as PLA and PLGA, have adjustable degradation properties, and their in vivo degradation products have no toxic effects on tissues [36]. In addition, synthetic materials with better mechanical properties can be designed using synthetic parameters. Bioceramics based on HA and tricalcium phosphate are advantageous in terms of their corrosion resistance and bioactivity. After implantation in the body, the materials gradually degrade through solution-driven and cell-mediated processes and are eventually replaced by newly-generated lamellar bone tissue [37]. However, their poor toughness and extremely high rigidity [38] also affect their role in bone defect repair. Generally, titanium, magnesium, and zinc are the metallic materials used for bone repair. Titanium and its alloys are widely used as metal implants in orthopedic surgical treatments; however, their non-degradability and stress-masking effects limit their application in the bone repair material field [39]. Magnesium and zinc, biodegradable metals which are necessary for maintaining normal life activities in the human body, have been confirmed in many studies to have good biocompatibility with human cells and tissues, attracting extensive attention from researchers [40].

Common artificial bone replacement materials currently available in the market, such as hydroxyapatite bioceramics, titanium alloys, and PLA, have disadvantages such as slow degradation, insufficient osteogenic activity, poor osseointegration ability, and poor strength, which make it difficult to meet the demand for bone defect repair [41,42].

### 2.2. Ideal Material for Bone Defect Reconstruction

The development of artificial bone repair materials with nano-micron multi-level bionic structures, good mechanical support properties, and osteoinductive and vascularization activities similar to those of natural bone is an urgent problem to be solved for bone defect treatment. The ideal bone repair material should match the bone defect, have good plasticity and biomechanical strength, excellent biocompatibility, osteoinduction and vascularization ability, suitable in vivo biodegradability, non-toxicity and non-immunogenicity, and capable of becoming a “bridge” for bone tissue regeneration after implantation to promote bone tissue growth and remodeling. Only in this manner can the requirements for bone defect reconstruction be satisfied. The best way to solve this problem is to mimic the composition, structure, and function of natural bone.

Compositionally, nano-HA and collagen fibers are basic components of natural bone, and their internal calcium–phosphorus system and the richness of trace elements such as Mg and Zn greatly improve their biocompatibility and biological activity [43]. As the basis of human biomineralization, calcium–phosphorus system materials have many applications and can play a regulatory role in stem cell osteogenic differentiation [44]. A review article by Surmenev et al. [45] showed that calcium–phosphorus coatings on the surface of materials have a significant effect on the bone regeneration process by enhancing cell adhesion, proliferation, and differentiation to promote bone regeneration. Liu et al. [46] showed that calcium–phosphate bone cement scaffolds with a calcium–phosphorus system enabled embryonic stem cells to survive and adhere, promoting new bone and blood vessel production. Other studies have shown that the calcium–phosphate system enables the regulation of cellular osteogenic differentiation. The ideal material for bone defect reconstruction should have a calcium–phosphate system similar to that of natural bone [47,48].

Structurally, the highly interoperable 3D porous structure and rich 3D capillary network inside natural bone are transport channels for nutrients and metabolites. The topology of this microenvironment and vascular network is conducive to stem cell adhesion, proliferation, and differentiation [49]. An ideal material should also have a highly interoperable pore structure with appropriate internal porosity for nutrient transport, metabolic circulation, cell migration, and permeability [50]. The structural characteristics of the ideal repair material are important for bone defect treatment. Tang Daniel et al. [51] suggested that improving the physicochemical properties of the material itself is also a way to reduce bone repair material limitations and to effectively promote bone regeneration. A large body of literature suggests that materials that promote bone regeneration when treating bone defects should have physicochemical properties similar to those of natural bone. Many researchers of bionic natural bone consider building materials at the microtopographical level [50,52].

Functionally, the natural bone matrix contains a variety of growth factors, including transforming growth factors, bone morphogenetic proteins, and fibroblast growth factors [53]. In the human body, they can induce differentiation of bone marrow mesenchymal stem cells to osteoblasts, and when the human skeletal system is damaged, these growth factors will be stimulated by body signals to release and aggregate toward the site of bone defects, thus playing a role in promoting bone healing growth and accelerating the repair process during the bone defect repair. The materials used to make the bone regeneration scaffold should be selected from artificial bionic substances that have similar effects to those of growth factors in natural bone, which can play a role in both the mechanical support and accelerated wound healing of the bone tissue during bone defect recovery.

### 2.3. Preparation and Effects of Bionic MC

MC has the composition and multi-level structure of natural bone, is biocompatible, and supplies a good and suitable microenvironment for the expression of bone cell activity after implantation; it is completely degraded and subsequently resorbed as bone tissue regenerates, crawls, and replaces, making it one of the best bionic materials available for bone defect repair [54,55]. 

**Figure 1:**
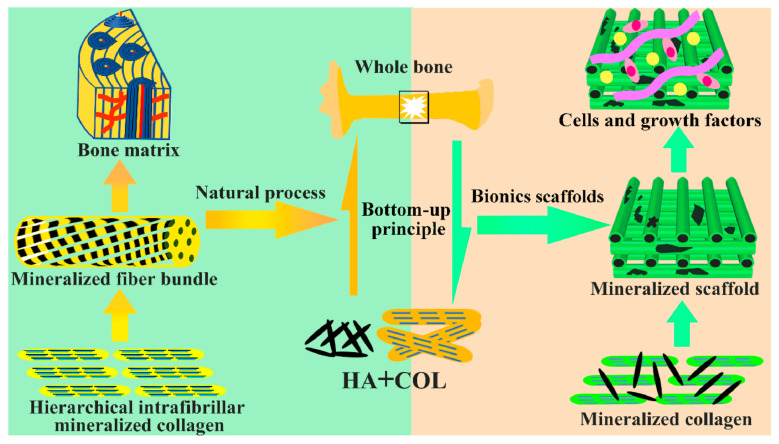
The natural bone formation process and the bionic strategy of a bone repair scaffold. The new bone formation process follows a bottom-up principle. The hierarchical intrafibrillar mineralized COL are formed by HA and COL and serve as a substance for the next level, which is an important manifestation of the bone hierarchical structure from micro to macro. The hierarchical intrafibrillar mineralized COL undergoes rearrangement and assembly to form fiber bundles. The mineralized fiber bundle is the basic unit of the bone matrix and provides the necessary spatial structure for bone formation. The bionics scaffold is fabricated by simulating the process of natural bone formation and provided a suitable microenvironment to promote bone repair. Used with permission under the CC BY-NC-ND license from reference [55]. Copyright © 2023 [The Author/The Authors], Zhengwei Li et al.

The basic structural unit of natural bone tissue is a self-assembled collagen fibril dominated by Col I, with HA nanocrystals embedded within the fibrils and the interfibrillar zone [56]. Biomineralization is a continuous process. In the initial stage, hydroxyapatite is deposited in collagen fiber voids, thus forming intrafibrillar mineralization. At a later stage, it is deposited on fiber surfaces, thus forming extrafibrillar mineralization. Collagen fibers have nucleation sites for apatite crystal particles, which allow them to guide the growth of mineral crystals and align them in a certain direction [57,58]. The mineralized fibers are then further bound, resulting in a highly ordered stacked structure, where Col I and HA are present in the bone in a nested, helical fashion [55] and form a dense or spongy bone. MC preparation is a nucleation process with a mineralization mechanism similar to that of natural bone, both with organic matter as the matrix and through inorganic phase nucleation [59]. The mineral phase, collagen, noncollagen, proteoglycan, and water in natural bone form an ordered multi-level structure on its surface under the action of various inducing factors, such as biomacromolecules and noncollagen proteins [60]. However, in the preparation of MC, its structure and function are influenced by its mineralization mode and the ratio of nano-HA to Col I. In extrafibrillar MC, HA crystals accumulate in a disorderly manner around the collagen fibrils, do not form an ordered microstructure, and do not have the same surface morphology and nanostructure as the bone extracellular matrix [61]. However, mineralization within the fibrils resulted in a better collagen bionanostructure. Better osteogenesis is generally achieved when the ratios of nano-HA and Col I are close to those of natural bone. Ou et al. [62] conducted in vivo and in vitro studies on four composite scaffolds with different HA/Col I mass ratios. The experimental results showed that scaffolds with an HA/Col I mass ratio of 7:3 had strong cell adhesion properties and were more osteoinductive. Sun et al. [63] also found that in material with an HA/Col I mass ratio of 7:3, the nano-HA particles were uniformly distributed on the collagen matrix pore walls, resulting in a stable and homogeneous porous structure. Figure 1 shows the natural bone formation process and the bionic strategy of a bone repair scaffold.

Collagen and nano-HA were assembled into mineralized fibers, based on which calcified collagen fiber bundles were assembled in parallel to form a hierarchical structure and porous system similar to natural bone. The literature suggests that Col I can enhance the biological activity of HA scaffolds [64]. MC has a pore size and rate similar to that of cancellous bone [65], which can provide good penetration of cells, new bone, and microvessels. In addition, MC can improve cell adhesion and migration ability, enhance angiogenesis, and promote osteoblast differentiation, providing a suitable cellular microenvironment for bone regeneration [66]. Therefore, MC has very good biological properties and is a promising material for bone defect repair. However, the current mechanical properties of a single MC are poor and cannot meet the clinical requirements of high-strength MC. PLGA has been widely used in the field of renewable materials because of its excellent mechanical properties, biocompatibility, and plasticity [67], and its introduction can reasonably regulate the pore size characteristics under the microstructure and provide buffering to the weak alkalinity of hydroxyapatite due to the release of calcium and phosphorus ions during the degradation process. The introduction of hydroxyapatite buffering during degradation due to the release of calcium and phosphorus ions maximizes implant material biocompatibility and improves its mechanical strength [68].

### 2.4. Advantages and Physicochemical Properties of PLGA

PLGA consists of two monomers, lactic acid and hydroxyacetic acid, polymerized in a certain ratio, without the presence of functional side groups; alternatively, a random copolymer, an amorphous polymer, is a degradable functional polymer organic compound with good biocompatibility, nontoxicity, and good performance in capsule and film formation. PLGA has been widely used in many fields, such as pharmaceutical, modern, and medical engineering materials, has passed strict U.S. FDA certification, is also officially included as a pharmaceutical excipient [17], and has now become an important class of biomedical polymer materials.

The internal form of PLGA is generally a linear copolymer [21], in which the two constituents have different linkage ratios, which is commonly referred to by its internal composition. PLGA is a light yellow or colorless substance with different properties depending on the synthesis method, and its composition ratio can also lead to changes in its properties. The ring-opening polymerization method is used by most researchers to prepare PLGA. This method uses two substances, lactic acid and glycolic acid, and the first step is dehydration cyclization to synthesize the two monomers, ethylene glycolate and propylene glycolate; thereafter, following ring-opening polymerization, a PLGA random copolymer is obtained. The internal composition of random PLGA copolymers can be controlled by different feeding ratios, and the ring-opening co-polymerization reaction can be performed in either the native state or in solution. In native polymerization reactions, the reaction temperature requirement is high, typically above the melting point of the monomer, whereas in solution polymerization, the temperature requirement is lower. The pressure required for the ring-opening copolymerization reaction is usually 1–10 mm Hg and requires purging with dry gas.

Although PLGA has good biodegradability, its rate of degradation can be artificially controlled to some extent [34]. The degradation of PLGA is generally uncomplicated and requires two distinct phases: lysis and uptake. In aqueous environments, enzymes released by microorganisms or cells are present on the surface of the PLGA polymers, facilitating this reaction. The hydrolysis reaction occurs on its internal ester bonds [69], and the large molecules are gradually broken because of the chains, leading to the formation of low-molecular-weight polymers, at which point the original mechanical strength is lost. When the molecular weight is reduced to the solubility limit in water, the overall structure of the substance changes, and deformation and weight loss will occur. In the next stage, tiny water-soluble fragments will gradually enter the human bodily fluids, followed by the decomposition of low-molecular-weight intermediates that are shed and dissolved in the human body, which can break down the metabolism of lactic acid, α-hydroxyacetic acid, and finally into carbon dioxide, water, and other end products. In this process, the hydrolysis of the ester bond in the polymerization chain of the substance is the fundamental cause of degradation. Simultaneously, the end carboxyl groups have a catalytic effect on the process, and the number of end carboxyl groups increases as the degradation process is prolonged, accelerating the degradation rate. In addition, the temperature, pH, molecular weight, molecular configuration, composition ratio, and geometry affect the PLGA degradation rate. The composition ratio has the greatest influence on the copolymer, with different ratios, crystallinities, water absorption, and hydrolysis rates [70].

**Figure 2:**
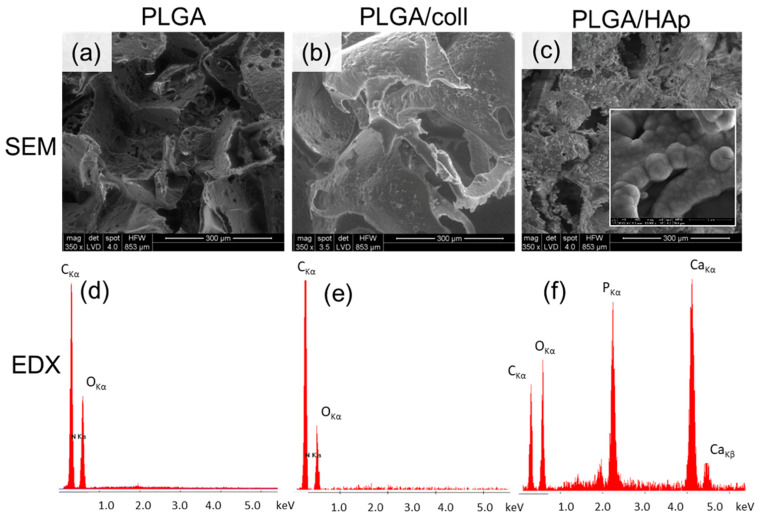
Microstructure by scanning electron microscopy (SEM) (**a**–**c**) and elemental analysis by energy dispersive X-ray spectroscopy (EDX) (**d**–**f**) of the scaffolds:Poly(l-lactide-co-glycolide) (PLGA) (**a**,**d**), Poly(l-lactide-co-glycolide) modified with collagen type I (PLGA/coll) (**b**,**e**), and Poly(l-lactide-co-glycolide) modified with hydroxyapatite (PLGA/HAp) (**c**,**f**). Insert in PLGA/HAp (**c**) shows globular cauliflower mineral deposits typical for low-crystalline hydroxyapatite. Used with permission under the Creative Commons Attribution (CC BY) license from reference [71]. Copyright © 2023 by the authors, Małgorzata Krok-Borkowicz et al.

**Figure 3:**
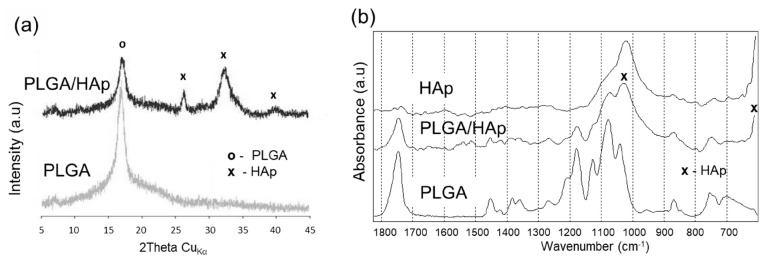
X-ray diffractometry (XRD) patterns (**a**) and Fourier transform infrared (FTIR) spectra (**b**) of PLGA and PLGA/HAp scaffolds. (**a**) and bands (**b**) marked by “x” are characteristic for hydroxyapatite. Used with permission under the Creative Commons Attribution (CC BY) license from reference [71]. Copyright © 2023 by the authors, Małgorzata Krok-Borkowicz et al.

The introduction of PLGA into bone repair materials can not only reasonably adjust the pore characteristics under the microstructure, optimize microenvironment topology, and provide transport channels for blood vessels, osteoblasts, nutrients, and metabolic wastes, but can also have a buffering effect on the weak alkalinity presented by the release of calcium and phosphorus ions during HA degradation, whereas PLGA as a polymer introduced into the repair materials can enhance implant material mechanical strength and biocompatibility. Figure 2 shows that PLGA bound well to collagen and hydroxyapatite, forming suitable pores. Figure 3 shows that PLGA has a functional group and structure similar to that of HAP.

### 2.5. Magnesium Doping Enhances Artificial Bone Repair Material Bioactivity

Mg^2+^ has good biocompatibility as well as anti-inflammatory, osteogenic, and angiogenic properties [72]. Mg is one of the main elements that make up bone, with approximately 50–60% of Mg^2+^ stored in the bone [73], and bone regeneration involves regulation of the bone environment, which is dynamic and constantly changing. Therefore, the stability and formation of the bone environment and Mg^2+^ are closely related [74]. Mg maintains bone strength, promotes bone formation, and prevents osteoporosis [75]. Mg^2+^ is gradually released during bone resorption, and the consequences of Mg^2+^ deficiency are more serious, resulting in accelerated bone loss and reduced bone formation, which can indirectly lead to stagnation of the bone regeneration process.

Mg^2+^ is generally recognized as the fourth most abundant element in the body and the most abundant divalent cation in cells. Mg^2+^ regulates various cellular functions including cell signaling, growth, metabolism, and proliferation [63]. Some studies have reported that biodegradable Mg^2+^ promotes the osteogenic differentiation of mesenchymal stem cells (MSCs) by enhancing autophagic activity [76]; however, the biological mechanisms are not fully understood [77]. The appropriate Mg^2+^ concentrations can activate the calcium channels in the cell membrane and promote calcium deposition. In addition, the angiogenic and anti-inflammatory functions of Mg^2+^ play special roles in bone regeneration conditions [78,79].

**Figure 4:**
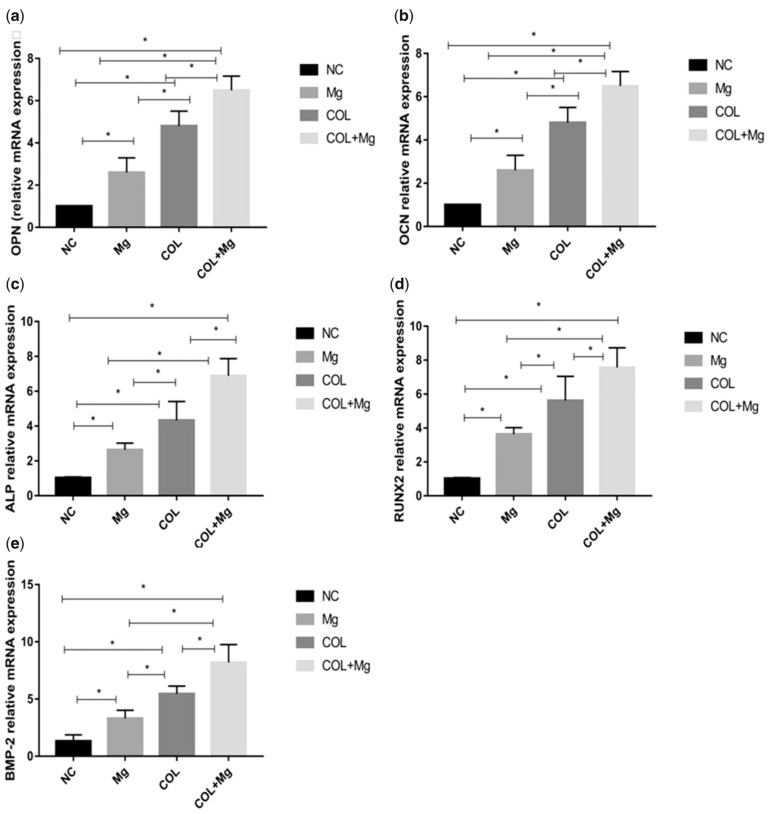
The NC (control group), Mg (10 mM Mg^2+^ group), COL (Col I-coating group), and COL + Mg (10 mM Mg^2+^/Col I-coating group) that osteogenic-related gene expression levels of OPN (**a**), OCN (**b**), ALP (**c**), RUNX2 (**d**), BMP-2 (**e**) at 7 days were quantified by real-time PCR. One-way ANOVA (*n* = 3 per treatment group). * *p* < 0.05 versus control group. Used with permission under the Creative Commons Attribution License from reference [80]. Copyright © The Authors 2019, Xiaojing Nie, et al.

Mg^2+^ promotes the conversion of macrophages to the M2 phenotype, and M2 macrophages secrete growth factors, including transforming and vascular endothelial growth factors. Suitable Mg^2+^ concentrations can promote migration and osteogenic stem cell differentiation. In addition, Mg^2+^ can promote the upregulation of the osteogenic genes, BMP2 and VEGF (Figure 4). Certain Mg^2+^ release concentrations can overcome the harmful immunomodulatory properties of Mg-based biomaterials, making them more favorable for bone marrow growth. Overall, Mg^2+^ induces an anti-inflammatory environment and promotes osteogenic differentiation. Therefore, controlling the concentration of Mg-based bone scaffolds could yield biomaterials with good bone immunomodulatory properties. These Mg ion functions lay the foundation for their widespread application in bone repair.

**Figure 5:**
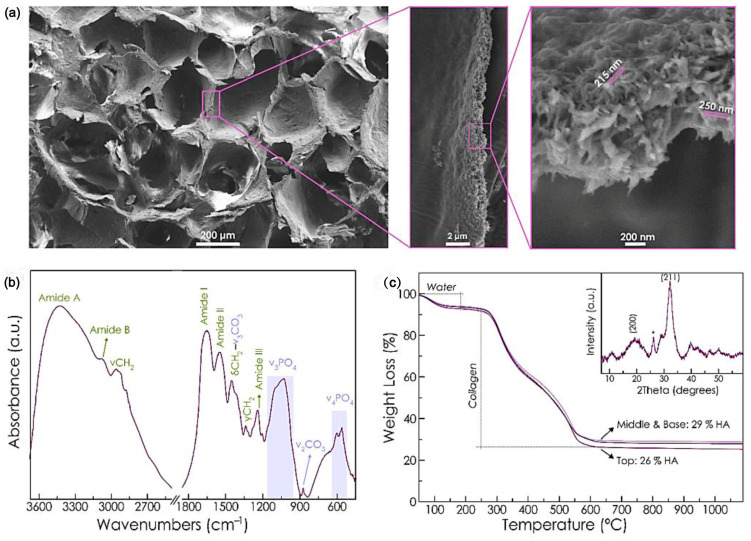
(**a**) SEM images of cross-section of RCP.Mg Ap scaffold. High magnification image of the wall of the pores shows the incorporation of Ap into the organic matrix. (**b**) Fourier transform infrared spectroscopy (FTIR) spectrum of RCP.MgAp scaffold. (**c**) TGA curve of the top, middle, and base of the scaffold. Inset displays X-ray diffraction (XRD) pattern of the sample exhibiting characteristic reflections of hydroxyapatite (HA, ASTM card file No 09-432). Used with permission under the Creative Commons Attribution (CC BY) license from reference [81]. Copyright © 2023 by the authors, Gloria Belén Ramírez-Rodríguez, et al.

Bionic MC in situ composite Mg^2+^ can change the size, density, nanostructure, and crystallinity of HA particles, increase the tensile strength and cell proliferation rate of the material, and improve osteogenic activity by upregulating the expression of RUNX22 and alkaline phosphatase [71]. The expression of osteogenesis-related gene sequences increased in MC3T3-E1 cells cultured with magnesium ion/type I colloidal material. The magnesium-doped bionic mineralized recombinant collagen scaffold exhibited a honeycomb pore structure and bound well to HA (Figure 5).

High levels of Mg^2+^ inhibit osteoblast proliferation and differentiation, and Mg^2+^ introduction becomes key for the bionic MC in situ composite Mg^2+^ scaffold to function [80,82]. It is a major challenge to improve the MC synthesis method to achieve the optimal content of functional trace element Mg^2+^ loading without changing the overall bionic structure and to improve the biological activity of Mg–MC/PLGA bone repair materials.

### 2.6. D Printing Technology for the Preparation of Bone Repair Materials That Mimic Multiple Dimensions of Chemical Composition, Hierarchical Structure, and Mechanical Properties

**Figure 6:**
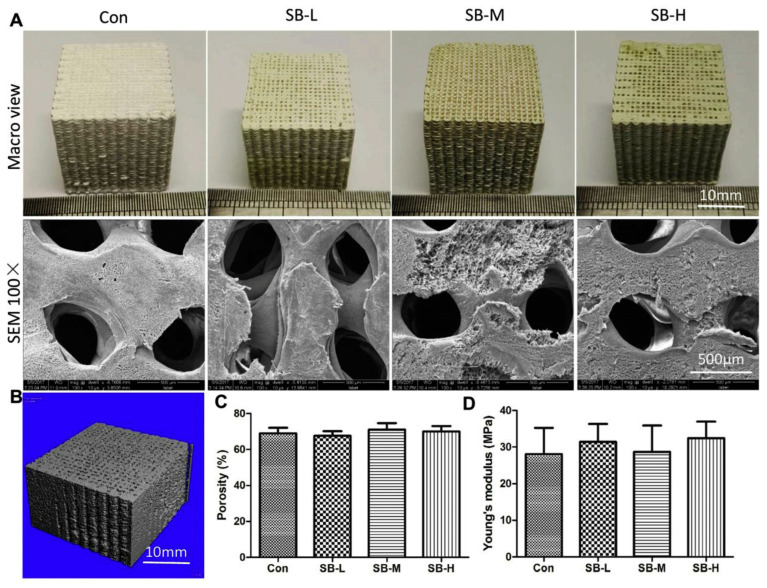
Macrograph image and physical properties of the fabricated PLGA/β-TCP scaffolds incorporated with none (Con) or with low (SB-L), medium (SB-M), or high (SB-H) dose of SB. (**A**) Macrograph and SEM images of the final product by 3D printing; (**B**) Representative image of microstructure of the scaffolds analyzed by microCT; (**C**) Porosity of the scaffolds measured by ethanol immersion method; (**D**) Young’s modulus as measured by mechanical testing. Data were shown as mean ± SD (*n* = 3). Used with permission from reference [85]. Copyright © 2023 Elsevier Ltd.

3D printing technology was first invented at the end of the twentieth century; when it was first introduced, it was more often used in industrial production and aerospace model manufacturing. With the continuous progress of science, 3D printing technology has been continuously optimized, many other functions have been gradually developed, and the application of this technology is also gradually expanding [83]. The application of 3D printing technology in medicine has attracted the attention of scientists worldwide. Considering the past history of medical development, human organs and tissues are unique and irreplaceable, and the means to extend human life by replacing organs and tissues is a global technical problem. However, with the rapid progress in life sciences and the continuous development of 3D printing technology in recent years, its use to create organs and tissues to extend human life has become a reality [84]. Assuredly, the increasingly mature 3D printing technology is bringing unprecedented changes to medicine. Figure 6 shows the 3D-printed PLGA/β-tricalcium phosphate scaffold.

Because of the complex structure and function of human organs, attempts to use 3D printing technology to create tissues and organs have begun with the more homogeneous structure of bone tissue. In 2009, the Third Hospital of Peking University conducted research on the use of 3D printing technology to manufacture artificial metal prosthetic bones. Using 3D printing technology, metal powder can be used to create prosthetic bones of any desired shape according to various complex bone defects. This is a unique advantage of 3D printing technology, and the finished product can be obtained within a few hours of modeling [86]. In addition, the bone created by 3D printing technology allows space for real bone growth in advance, and as the treatment time increases, human bone and implanted prosthetic bone can be integrated to maximize patient recovery. Although the technology of using alloy materials to make human prosthetic bones is relatively mature, the exploration of using other materials to make human bones continues, and the current research direction of artificial bones is increasingly moving toward biocomposites [39], which can provide passive porous structures while supporting or guiding bone formation.

To fabricate Mg–MC/PLGA scaffolds using 3D printing technology, mimetic Mg–MC fibers with a highly similar chemical composition and micro-nanostructure to natural MC were prepared under physiological conditions to achieve a fine self-assembly of collagen microfibrils and nano-HA crystals to achieve mimicry on the micro-nanoscale. Porous Mg–MC/PLGA bone repair materials mimicking cancellous bone and dense materials mimicking cortical bone were prepared at the micromillimeter scale using low temperature deposition bio-3D printing technology. Subsequently, at higher scales, advanced biomimetic bone repair materials with dense/porous biphasic composite structures providing both strength, support, and induced osteogenic activity were prepared. A study has been conducted to apply the cancellous bone mimetic Mg–MC/PLGA material to treat cancellous bone defects in rabbit femoral condyles. The imaging, histological, and biomechanical performances of the experimental group were found to be significantly better than those of the blank group at 4, 8, and 12 weeks postoperatively, and there was no significant difference between the two groups. For the first time, the efficacy of Mg–MC/PLGA in treating smaller defects in long bones was demonstrated to be consistent with that of autologous bones. On this basis, concentrate growth factors (CGF) was added using a physical mixing method to release growth factors that promote the proliferation of osteoblasts and formation of vascular tissue in the early postoperative period [87], and to be absorbed by bone tissue in the middle and late periods to increase the nutrient supply and active factor boosting during the bone regeneration and repair process [88,89].

## 3. Current Status of Research on MC/PLGA Scaffold Materials for Bone Defect Treatment

**Figure 7:**
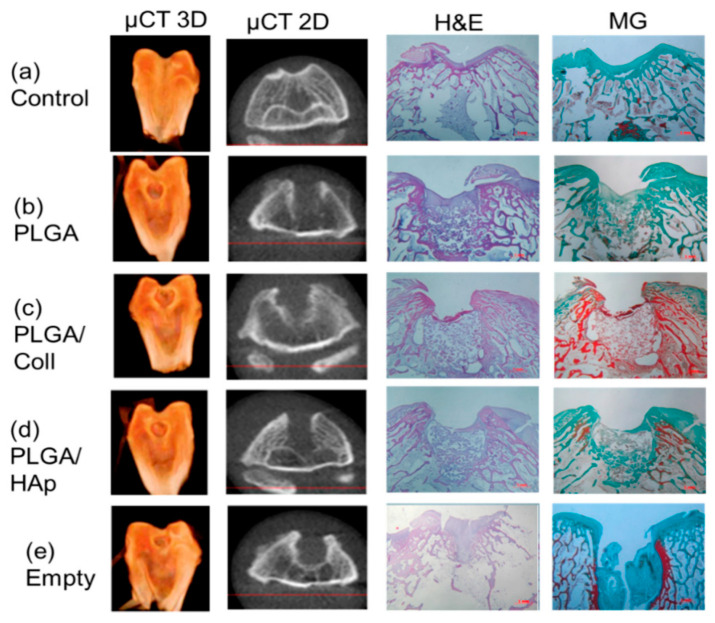
Computer tomography in 3D and 2D projection (first and second column) and histological evaluation after hematoxylin and eosin (third column) and Masson–Goldner staining (fourth column) 4 weeks post-implantation: (**a**) control, (**b**) defect filled with PLGA scaffold, (**c**) defect filled with PLGA/coll scaffold, (**d**) defect filled with PLGA/HAp scaffold, and (**e**) empty defect. Used with permission under the Creative Commons Attribution (CC BY) license from reference [71]. Copyright © 2023 by the authors, Małgorzata Krok-Borkowicz et al.

MC/PLGA scaffolds are new bone repair materials composed of MC and PLGA prepared using in situ mineralized bionanotechnology with good biocompatibility, osteogenesis, degradability, and mechanical properties [89,90,91,92]. Natural bone is a mineral/organic natural complex material composed of hydroxyapatite and collagen; therefore, MC-based materials prepared using calcium phosphate and collagen are the best biomimetic bone substitutes [33,93]. Its physical properties are similar to those of natural bone, with a micron/nanoscale structure close to the extracellular matrix of natural bone tissue, which provides structural support and a cellular microenvironment for cell growth [93]. MC/PLGA materials have been shown to contribute to enhanced early cell adhesion and proliferation with osteogenic and chondrogenic properties [71,94,95,96,97], as shown in Figure 7. Further studies have shown that the scaffold pore size affects cell growth and differentiation, with large pore size scaffolds being more favorable for MSC differentiation and nanoscale scaffolds being more favorable for cell proliferation [98].

**Figure 8:**
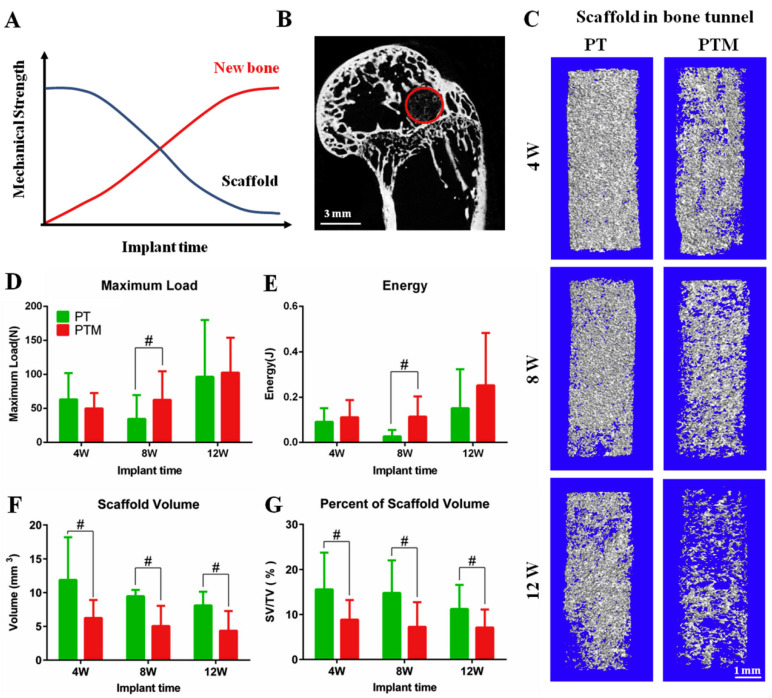
Compression tests and in vivo scaffold degradation. (**A**) Ideal degradation behavior of scaffold in vivo accompanies with bone defect repair. (**B**) 2D micro-CT images of the defect site used to guide the biomechanical tests. The red circle shows the positioning the bone tunnel. (**C**) The residue of scaffold in bone tunnel at each time point from PT and PTM group. (**D**,**E**) Biomechanical tests results. At 8 weeks after surgery, the maximum load, and the energy in the PTM group were significantly higher than those in the PT group (*p* = 0.02, *n* = 4). (**F**,**G**). The changes of scaffold volume during the degradation after surgery in each group. ^#^ *p* < 0.05 vs. PT group. Used with permission from reference [100]. Copyright © 2023 Elsevier Ltd.

The mechanical properties of single MC are poor and insufficient to satisfy the high-strength demand for bone repair. The introduction of PLGA adjusts the pore characteristics under the MC microstructure and enhances the mechanical strength of the material to satisfy the supporting role of the bone repair material [99]. In addition, dynamic structural properties prevent stress masking during bone repair. It has been shown that the scaffold material degrades slowly as new bone tissue grows in, and the biological stress is gradually transferred from the scaffold to the bone tissue so that the bone tissue receives sufficient mechanical stimulation during the rehabilitation process [6]. Figure 8 shows that a material containing a magnesium composite, PLGA/tricalcium phosphate, can effectively promote bone defect healing with good mechanical properties and degradation.

MC/PLGA scaffold biological properties for repairing bone defects can be improved by loading them with growth factors or drug components. Bone morphogenetic protein-2 is a common osteogenic inducer involved in the direct differentiation of mesenchymal stem cells into osteoblasts [72]. BMP-2 can improve the bone regeneration ability of MC/PLGA materials [101,102]. Insulin, usually used as a hormone and drug to maintain energy and glucose stability, has a positive effect on bone marrow MSC adhesion and proliferation as well as osteoblast differentiation when loaded into MC/PLGA scaffolds [103]. Open fractures are prone to infection complications, and infected bone defect repair is a great challenge in clinical practice. The development of bone defect scaffolds with antimicrobial properties is particularly important. He et al. [104] prepared bone scaffolds with antimicrobial properties by encapsulating antimicrobial peptides in PLGA microspheres and embedding them in MC scaffolds; their results showed good local anti-infective and osteogenic activities. Vancomycin, a common antibacterial drug, can be delivered to the fracture site through MC and PLGA scaffolds to repair bone defects while inhibiting infection, and the mechanical properties of the material can be changed by controlling the crystallinity of PLGA to meet different patient requirements [105]. Liu et al. [106] prepared PLGA nanofibrous bone scaffolds loaded with hyaluronic acid oligosaccharide–MC particles. The irregularly oriented nanofibers in this scaffold mimic the anisotropic microstructure of the natural bone matrix and evoke an early cellular response to bone repair by upregulating angiogenic gene expression.

## 4. Conclusions and Outlook

MC prepared using molecular self-assembly technology has a composition and hierarchical structure similar to that of natural bone. 3D printing technology can be used to assemble MC and PLGA into mock cortical and cancellous bone scaffolds with different pore structures. The MC/PLGA scaffold is an ideal bone-repair material with good biocompatibility, osteogenesis, degradability, and mechanical properties. Magnesium ion doping can improve scaffold osteogenic, angiogenic, anti-inflammatory, and biological properties for better application in bone defect repair by loading growth factors and drug components. Further experimental validation of MC/PLGA scaffolds doped with Mg ions for bone defect repair is required.

## Data Availability

No new data were created.
